# A qualitative study exploring children and young people's experiences of cognitive therapy for PTSD delivered within the context of a randomised controlled trial

**DOI:** 10.1111/papt.12558

**Published:** 2024-11-18

**Authors:** Leila Allen, Andrew Fox, Alexandre Copello, Richard Meiser‐Stedman, Nicola Morant

**Affiliations:** ^1^ Department of Clinical Psychology and Psychological Therapies, Norwich Medical School University of East Anglia Norwich UK; ^2^ Centre for Applied Psychology, School of Psychology University of Birmingham Birmingham UK; ^3^ Research and Innovation Birmingham and Solihull Mental Health Foundation NHS Trust Birmingham UK; ^4^ Division of Psychiatry UCL London UK

**Keywords:** children and young people, cognitive therapy, PTSD, qualitative research, thematic analysis, trauma

## Abstract

**Objectives and Design:**

This qualitative study aimed to explore the treatment experiences of children and young people with Post‐traumatic Stress Disorder (PTSD) symptoms, participating in a randomised controlled trial evaluating the effectiveness of Cognitive‐Therapy for PTSD (CT‐PTSD).

**Methods:**

Thirteen participants aged between 12 and 18 years old, who had all experienced multiple trauma and had undertaken CT‐PTSD, were interviewed.

**Results:**

Using thematic analysis, three key themes were identified: ‘*Desire for difference*’, ‘*Journey of becoming able to talk about trauma*’ and ‘*Positive changes and increased ability to cope’*. Prior to the study, participants described experiencing difficult emotions and avoided talking about their traumatic experiences. Participants reported wanting to get the right help and valued the opportunity to help others. Talking about trauma during treatment was perceived as difficult and emotionally draining, however participants reported a sense of relief and that it became easier over time, helping them to make sense of their traumatic experiences. This was facilitated by the therapeutic relationship, their involvement in decision making and the use of written tasks. All participants reported positive changes, both in themselves and in their ability to talk to others about their traumatic experiences.

**Conclusion:**

Engaging in CT‐PTSD and talking about traumatic experiences can be empowering for young people and allows them the opportunity to process their trauma leading to increased ability to cope.

**Practitioner Points**

- Young people with PTSD found that accessing Cognitive Therapy helped them to process their trauma, was empowering and reported positive changes in their relationships and ability to cope.
- Valued aspects of therapy included involvement in decision making and the use of written tasks (e.g. timelines).

## INTRODUCTION

Traumatic events, defined as exposure to actual or threatened death, serious injury or sexual violence (DSM‐5; American Psychiatric Association, 2013), are experienced by nearly a third of children and young people in the United Kingdom, with one‐fifth of them developing post‐traumatic stress disorder (PTSD) (Lewis et al., 2019). Furthermore, in a national survey in the United States of over 10,000 adolescents, 5% of them met criteria for PTSD in their lifetime (Merikangas et al., 2010). PTSD in ICD‐11 (World Health Organisation, 2022) comprises of three key symptom clusters: re‐experiencing the trauma, avoidance of trauma‐related stimuli, including trauma‐related thoughts and feelings, and increased arousal and reactivity from a persistent sense of threat. The National Institute for Health and Care Excellent (NICE, 2018) guidelines suggest that children and young people with PTSD are first offered trauma‐focused cognitive behavioural therapy (TF‐CBT), and only if they do not respond to or engage with this then clinicians should consider eye movement desensitisation and reprocessing.

Research suggests abuse and neglect in the United Kingdom is not uncommon (Radford et al., 2011), with between 4% and 16% of children per year experiencing physical abuse, and between 10 and 25% exposed to domestic violence (Gilbert et al., 2009). It is well documented that multiple traumatic experiences are likely to have a detrimental long‐term impact on children and young people's development and well‐being (Downey & Crummy, 2022). For some, the impact may be externalised, for example through their behaviour such as anger outbursts, or internalised, resulting in young people withdrawing from their environments and presenting with low mood (Bentovim et al., 2009).

It has been argued that young people's perspectives provide the most reliable source of information regarding their own experiences (Morrow & Richards, 1996). Whilst research on children's experience of trauma‐focused therapies is still limited, there are a number of emerging studies that shed light on how young people perceive therapy that have important clinical implications. In a qualitative study 30 youths between 11 and 17 years old were interviewed about their experience of Trauma‐Focused Cognitive Behavioural Therapy (TF‐CBT), where it was found that the majority found trauma exposure work helpful, despite initial concerns (Dittmann & Jensen, 2014). Participants considered important change processes to be acquiring new perspectives and learning skills for managing stress, informing clinical research about the best way to tailor therapeutic work for trauma‐exposed children and young people.

Similarly, in a study with 16 children, aged between 8 and 12 years who had all received TF‐CBT, it was found that children found the therapy helpful overall, with nine children valuing the trauma narrative activities (Salloum et al., 2015). Three of the children found the trauma narrative component unhelpful and wanted to avoid talking about the trauma. This suggests that further research is needed to explore youth's perception of trauma‐focused work and to understand the acceptability and feasibility of psychological interventions for PTSD to ensure that children and young people receive the most appropriate and effective treatment.

### Study setting and aims

Interestingly, more qualitative research is now emerging to help understand more about how young people make sense of their traumatic experiences, and furthermore their experiences of treatment (Ascienzo et al., 2022). Crucially highlighting how therapists delivering trauma‐focused interventions need to recognise the importance of being attuned to clients, recognising the difficulties of trauma work and promote self‐determination (Eastwood et al., 2021). This qualitative study aims to add to the literature exploring the experience of trauma focused interventions for children and young people.

This qualitative study was embedded within a UK randomised controlled trial (RCT) ‘DECRYPT’ (Delivery of Cognitive Therapy for Young People After Trauma; Allen et al., 2021). The primary objective of DECRYPT was to evaluate whether Cognitive‐Therapy for PTSD (CT‐PTSD) was an effective treatment for children and young people aged between 8 and 17 years old presenting with PTSD symptoms who had experienced multiple traumatic experiences, in comparison to treatment‐as‐usual provided by Child and Adolescent Mental Health Services (CAMHS). The intervention–CT‐PTSD–is a structured, manualised psychological treatment for children and young people with PTSD aiming to help young people form more coherent, elaborated memories of their trauma, restructure maladaptive appraisals, and discourage the use of maladaptive coping strategies (Smith et al., 2010). 120 children and young people were recruited to the RCT between February 2017 and June 2021. Both treatment arms were delivered by NHS CAMHS therapists (of any qualified background, i.e. nurse, clinical psychologist). Participants were recruited from four NHS sites across the United Kingdom.

The main inclusion criteria for the DECRYPT trial were young people who had experienced multiple traumatic events and were experiencing high levels of PTSD symptoms (as defined by scoring 17 or above on the Children's Impact of Event Scales, CRIES‐8, Horowitz et al., 1979) and met a diagnosis for DSM‐5 PTSD (DSM‐5; American Psychiatric Association, 2013). Full inclusion and exclusion criteria and further information regarding CT‐PTSD are detailed in the DECRYPT Protocol paper (Allen et al., 2021). It is worth noting the complexity of the young people involved in this study, with high levels of co‐morbidities including complex PTSD. This RCT was also embedded within routine clinical practice and therefore the findings represent the experience of young people in a more pragmatic, real‐world setting.

The current qualitative study explores the experiences of children and young people who received CT‐PTSD within DECRYPT, offering potentially important insights into the acceptability and feasibility of CT‐PTSD and potentially informing clinician attitudes to the management of trauma‐exposed children and young people.

## METHOD

### Ethical considerations

The clinical trial and associated qualitative evaluation were approved by The Cambridge South Research Ethics Committee (16/EE/0233, July 2016).

### Recruitment and participants

Participants who had received CT‐PTSD in DECRYPT were invited to participate in the qualitative study either during or following their 5‐month post‐treatment assessment. They were provided with an age‐appropriate information sheet and were given at least 48 hours to consider participation. The trial manager obtained verbal consent to pass on their contact details to the researcher to arrange the interview. In addition to the main trial consent form, a separate consent form was completed at the beginning of the interview to ensure that participants understood and agreed to participate, in particular that they consented to the interview being audio recorded and the use of anonymous quotes in publications. Parental consent was also obtained for young people under the age of 16 years.

### Data collection

A semi‐structured interview guide was developed with feedback from public and patient involvement (PPI) representatives, through regular meetings. Two interview guides were used, one for children up to 12 years old and one for children and young people over 12 years old. The PPI representative's views were key in ensuring that the language and structure of the interview was age appropriate.

Seven of the interviews were conducted by the first author (LA) and six were completed by trial managers, none of whom were involved in the youths' therapy or research assessments. In preparation for the interviews and to tailor the questions accordingly, the interviewers had information regarding the traumatic events the young people had experienced, whether there had been any serious adverse events (to establish if there was any clinical risk) and whether they had been discharged or were continuing to receive therapy.

### Data analysis

The interviews were transcribed by the first author (LA) and analysed according to Reflexive Thematic Analysis (Braun & Clarke, 2019), using NVivo version 12 software. Thematic analysis provides a flexible method to identify, analyse and report patterns or themes (Braun & Clarke, 2006), and is a useful method for examining perspectives of different research participants, highlighting similarities and differences, and generating unanticipated insights (King, 2004). The stages of analysis and examples are highlighted in Table 1.

**TABLE 1:** Stages of analysis taken in Thematic Analysis, guided by Braun and Clarke (2006).

Phase	Process
Phase one: Familiarisation with the data	Data was transcribed verbatim, reading and re‐reading the transcripts. Initial notes and reflections were detailed in a reflective journal.
Phase two: Generating initial codes	This phase involved line by line thematic coding, creating memos of interesting findings to review in later stages. Each transcript was coded using the software package NVivo version 12, allowing the author code and collate extracts of text. As an example of coding at this stage, the sentence ‘it was very useful, I found it really helpful’ was coded under ‘Thoughts about involvement in DECRYPT’. As advised by Braun and Clarke (2006) data was coded inclusively (i.e. some text surrounding the extract was included in the code) to capture the context of the code. Some extracts were coded multiple times if they captured a number of things, which ensured inclusivity. If the researcher was unsure what to code a particular data extract as, it was coded under ‘unsure’ and revisited when all other coding had taken place. Finally, all codes were reviewed and merged, when necessary (e.g. ‘became easier over time’ and ‘relaxed over time’ were merged to reflect the same code ‘easier over time’).
Phase three: Searching for themes	This phase involved looking for emerging themes where the codes could be grouped together to capture the most salient patterns in the data. For example, ‘feeling angry’ and ‘feeling upset’ were merged together to form ‘experiencing difficult emotions’.
Phase four: Reviewing themes	The fourth phase involved reviewing the themes and memos, some of the themes were expanded to include more codes, for example the code ‘more sociable’ was gathered under the ‘positive changes’ theme. Relationships between the themes were explored using ‘thematic maps’ which linked to the codes and were reviewed regularly with the research team.
Phase five: Defining and naming themes	The final themes and their structure were reviewed, defined and named. As a result of this process, some themes/subthemes were merged (e.g. ‘positive changes in themselves’ and ‘changes in relationships’ were merged to form an overall theme of ‘positive changes’).
Phase six: Producing the report	Finally, throughout the report a selection of compiling extracts were identified to demonstrate each theme and to illustrate the participants' experience.

The author took a critical realist epistemological stance to data analysis, recognising that as researchers we assume, although cannot ‘know’, a fixed reality, but we can understand participant experiences through close examination of their individual perspectives. A critical realist epistemological stance was adopted due to the main researcher's previous experience of working as a trial manager prior to completing the qualitative component of the study as part of a Clinical Psychology Doctorate thesis, therefore recognising that pre‐existing theory and knowledge was present. The analysis followed a primarily inductive thematic approach focusing on generating data driven analyses. After each interview reflections were captured in a journal to support researcher reflexivity particularly around interpretations of the interview data. Furthermore, for those interviews not conducted by the main researcher a meeting with the interviewer was arranged shortly after the interview had taken place to discuss any reflections. In addition, once the main researcher had listened to the audio recordings of the interview, feedback regarding the interviewers positioning and approach was offered to ensure consistency in future interviews. Lastly, to minimise the risk of bias and to improve the rigour of the analysis, the main researcher was provided with regular supervision with external supervisors, to reflect on their positioning throughout the data collection and analysis stages.

## RESULTS

### Participants

Thirty‐five eligible participants were offered the opportunity to participate in the qualitative interview, of which 13 agreed to participate (response rate 37%). Two of the eligible participants had dropped out of the research study and were contacted to ask whether they wanted to participate in the qualitative component of the study, however they did not consent to participate. Thirteen children and young people who had received CT‐PTSD were interviewed between July 2018 and March 2020.

Most participants chose to conduct the interviews at their home, whilst one interview was completed at an NHS site. All interviews were completed face‐to‐face, and efforts were made to ensure that the interview took place in a quiet environment, where interruption was minimised. One 12‐year‐old participant had their parent present for the interview. The interviews were completed approximately 10 months after the participants had been randomised (ranged from 6 to 15 months post‐randomisation) and the average length of the interviews was 50 min (ranged from 27 to 74 min).

The 13 participants were aged between 12 and 18 years old (*M =* 15.7, *SD =* 2), of which 10 were girls and three were boys (denoted by F or M for anonymised data extracts below; see Table 2). All participants had undertaken CT‐PTSD, ranging from 2 to 15 sessions (*M* = 9.2, *SD* = 4.6). To preserve anonymity types of trauma are not detailed for each participant, however, all participants had experienced multiple traumatic events which included the following: sexual abuse (*N* = 3), physical abuse (*N* = 2), emotional abuse (*N *= 2), domestic violence (*N* = 5), bereavements (*N* = 3), severe bullying (*N* = 5) and/or sexual assault (*N* = 2). Three participants were continuing to receive CT‐PTSD and 10 had been discharged from CAMHS.

**TABLE 2:** Demographic information about the participants.

Participant ID	Age
F001	18
F002	18
F003	16
F004	18
F005	16
F006	15
F007	15
F008	18
F009	17
F010	16
M001	12
M002	14
M003	12

### Summary of themes

**FIGURE 1:**
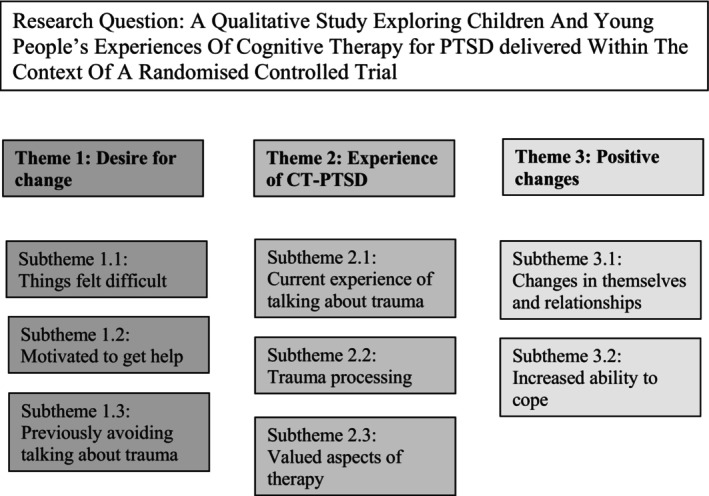
Research question and findings (themes and sub‐themes).

Three key themes were identified, each with a number of sub‐themes and are displayed in Figure 1.

### Theme 1: Desire for change

All participants expressed a sense of ‘desire for change’, describing everyday life as difficult prior to involvement in DECRYPT and wanting to get the right help in order to feel better.

#### Subtheme 1.1: Things felt difficult before involvement in DECRYPT

Nearly all participants spoke about experiencing difficult emotions prior to involvement in DECRYPT, in particular feelings of anger and upset. Ten participants, including all three males, described feeling angry and some alluded to this being the only way to express their emotions:

I would get angry a lot because that's the only emotion I knew –F006

Participants reported experiencing difficulties in everyday life, most commonly panic attacks, which were often related to experiences of reliving the trauma and increased hypervigilance. Participants also described experiencing regular flashbacks and nightmares, ‘*I kept on having, I was having nightmares a lot, of things that had happened’‐*F002, and many of the participants described how they were isolating themselves from others illustrated by F007 ‘*…I didn't want to be around people at all’*.

Most of the participants (*n* = 9) described difficulties at school including finding it difficult to concentrate, getting into trouble and underperforming. Overall, there was a sense that participants felt things were ‘*bad*’ and they were in a ‘*bad place mentally*’*–*F009, and that these difficulties had persisted over a long period of time.

#### Subtheme 1.2: Motivated to get help for themselves and to help others

When discussing reasons for being involved in DECRYPT, many participants (*n* = 10), described being motivated to get help for themselves in order to help with the difficulties they were experiencing. Whilst most participants had engaged with previous therapy or support, many of them reported it as unhelpful and only one participant had undertaken previous trauma‐focused intervention, which she described as a positive experience. Participants described having been on a journey to get help and a sense of eagerness to now get, and engage with, the right help:

I said yes, because I was game for anything at that point to try and help … I was kinda at the point where I was, where no‐one was helping so the one opportunity I had to get better, I leaped –F005

Half of the participants described altruistic reasons for being involved in trauma‐focused research for young people (i.e. wanting to help other young people who had experienced traumatic events). They described wanting to be involved in research to improve access to psychological therapies for other children and young people who had experienced traumatic events:

…just I'm glad that this could help, “something positive coming out of my negative experience” which is really good –F004

#### Subtheme 1.3: Previously avoiding talking about trauma

Participants described how prior to the study, they had found it difficult to talk to others about their traumatic experiences, including their family and professionals:

It was horrible because like I didn't want to speak to anybody, like even people I did know kinda thing I didn't want to speak to –F008

Linked to this, a few participants described how when they had spoken about their traumatic experiences with professionals in the past, they felt they had not been given further help and there appeared to be a sense of mistrusting professionals and avoiding talking about trauma again:

…because I think I felt I had spoken to so many different people, I don't like talking about it with too many different people because I have before, and they never really helped so I feel like, I think it's the fear of, me saying it then, them not really doing anything with that but feeling sorry for me there. I think that's what I don't like because when, sometimes when, they're like I'm really sorry that happened to you, that's awful, but then I don't get the help I feel like I need, I feel like that doesn't make me want to talk to them –F002

About half of the participants described previous psychological support as unhelpful, four perceived previous support as helpful, one participant was ambivalent, and one had not received any prior support. Of those who found previous support unhelpful, four participants experienced therapists/counsellors as ‘*out of their comfort zone*’ or ‘*not handling the situation*’. There appeared to be a consistent narrative in which therapists and counsellors seem to avoid talking about the traumatic experiences and focused on general anxiety, depression, self‐esteem and/or eating disorder:

…she was like “you need to boost your self‐esteem” and she didn't help with the flashbacks at all, and she just focused on my self‐esteem, and I was like that's not really important to me –F006

One participant described feeling previously let‐down by professionals and that her traumatic experiences had been dismissed and avoided by others. On the other hand, those participants who described previous support as helpful, even though the focus was not on their traumatic experiences, some reported short‐term benefit ‘*we learned to punch like take on like soft things like pillows, rather than like real people*’*–M003*.

To summarise, participants described a difficult journey prior to involvement with DECRYPT and there appeared to be a mixture of previous experiences of therapy that some perceived as helpful and some not. Overall, participants alluded to the study *‘coming at a good time*’ and appeared to express a desire for something to be different, both with regards to how they felt in themselves and what they wanted from psychological therapy.

### Theme 2: Experience of CT‐PTSD

This theme relates to the experience of participants becoming more able to talk about their traumatic experiences through therapy over time, including what this experience was like and which aspects of the therapy they valued.

#### Subtheme 2.1: Current experience of talking about trauma

Participants spoke about the differences between CT‐PTSD and previous therapy, suggesting they felt that their current therapy targeted their difficulties, ‘*you know like really going over my issues and things like that, I've never really had to do that before, and I definitely feel like it made an impact*’–F001. In addition, one participant discussed how her therapist was able to help her tolerate difficult emotions, rather than avoiding them:

Because previously if I thought I was getting upset, I didn't like getting upset but now I've more‐ I've learned more just to deal with them feelings, instead of just pushing them away so it's just a completely different technique to what people used to do before, like kinda, distracting and putting away because I was getting upset –F002

When asked what it had been like talking about their traumatic experiences, six participants indicated that their initial reluctance to talk about their experiences reduced over time:

At the beginning I didn't really like it, because I didn't, like having to speak to someone I didn't know kind of thing, and then after a few sessions I started feeling a bit better and started opening up a bit more –F008

Participants described talking about trauma as emotionally draining (*n* = 4), with one participant describing how she would have to sleep after therapy sessions. When describing what it had been like talking about trauma, seven participants alluded to a sense of relief, illustrated by F003 ‘*in that way it was like a weight lifted off my chest*’. Furthermore, participants recognised it was difficult but consequently felt relieved.

When reflecting about the experience of therapy, three participants described feeling proud of themselves for talking about their traumatic experiences and alluded to this being an achievement, discussing how they felt they had taken ownership over their thoughts:

I feel very, very accomplished because by the time we ended the therapy I had processed everything that had happened, and I understood how things happened which has helped me so much when, thoughts of the trauma come back, and erm, kind of get me down because then I could (…) knowing all of the facts, I can just, kind of, battle those thoughts out of my brain, and then I am okay again –F010

In summary, the participants were very positive about their experience of CT‐PTSD. Most participants identified that initially it felt difficult to talk about their traumatic experiences, however it became easier over time and resulted in feeling relief and a sense of achievement.

#### Subtheme 2.2: Trauma processing

Participants described talking about their trauma as difficult but important and necessary in order to be able to process it and move forward (*n* = 6):

That was, it was difficult, but at the same time it was, erm, kind of enlightening, I guess. It kind of made me realise, the dictatorship that was held over me for such a long time, and it kind of helped me to move on from it –F009

Three participants described how encouragement from their therapist helped them to move forwards:

And it came from [therapist name] in the sense of that, it wasn't like a kick, it was sort of like a nudge, like “you're going to feel better someday” like if even if you don't feel like that's going to be now, like it will happen one day, and you've just got to wait –F006

Despite the reliving work feeling difficult, eight participants described how the reliving work helped them to make sense of their traumatic experiences and that it helped them to accept what happened:

It was more like helpful in a way, because everything, different things were talked about and sorted and erm, not just really with her, but also myself, I kind of figured out like, um, like that things need to be moved on from and I can't let them kind of like take over my life and stuff –F009

Further evidence of trauma processing was provided by participants who described how talking about the trauma in detail with their therapist had reduced the time they were now thinking about their traumatic memories:

Talking about it is getting it out of your body so like, you can be thinking about it in your mind, but if you talk about it, it won't be just sitting there –F007

Lastly, 10 of the participants described a reduction in symptoms, such as flashbacks and nightmares, and whilst some described that their flashbacks or nightmares had completely stopped, some of the participants reflected that these still occurred, but that they understood them better, their frequency and intensity had reduced, and they felt more able to manage them when they did happen.

Yeah, I would definitely say I'm handling my symptoms a lot easier, erm, getting less of them, less what's the word? Not frequent, but like intense –F001

Furthermore, it was evident from three participants that there was reduction in self‐blame as they described blaming themselves less for their traumatic experiences:

[when asked what he had learnt in the sessions] ‘erm, that what happened wasn't my fault’ –M003

#### Subtheme 2.3: Valued aspects of therapy

There was a strong sense that participants valued therapist flexibility and encouragement to take ownership and control over the therapy sessions. It appeared that the participants found being in control of the pace of talking about their traumatic experiences helpful, for example choosing which traumatic memories to focus on and being given the option to take breaks if they felt they needed to, ‘*she's like you can have breaks in between, and like, breaking it down and we can do it when you're ready, yeah*’*–*F003.

Participants also expressed that it was important to be involved in the decision‐making, for example having choice over completion of homework tasks, the gender of their therapist and the setting where they spoke about their traumatic experiences. When discussing homework, whilst some reported finding homework tasks helpful, several participants did not. Notably, it appeared that participants felt it was important to be given a preference about whether to complete homework or not:

I think it's also mainly because she's given me a choice to do them [homework tasks], like I've never felt forced to do them because I think when you feel like you have to do something, you don't really want to do it, especially because they're related to the sessions as well it's like I want to do them, so things like, when she said to me looking at things, I think I had a list and I could choose what I wanted to, that one was particularly helpful – F002

With regard to the setting, some participants described feeling more comfortable talking about trauma in their home environment, describing how home felt like a ‘*safe place*’ which helped them to open up. On the other hand, some young people described having a strong preference for therapy taking place in a clinic setting highlighting the importance of individual preference on how and where trauma‐focused work takes place.

…like when I leave that room it feels that that was all left in that room and I can kind of, carry on with things more –F002

Another facilitator to talking about trauma was the use of written tasks, which were highly valued by participants. Several participants referred to finding the timeline of their traumatic events a particularly helpful task (*n* = 6), reflecting that it helped to make sense of what happened and facilitated the processing of their trauma. The use of the timeline appeared to provide another opportunity for the young people to control the pace of the therapy sessions, participants describing how they could choose to focus on the ‘lighter memories’ before building up to working on the more challenging ‘heavier memories’.

In addition, one of the youngest participants provided an example of creative expression regarding his traumatic experiences saying ‘*because I didn't know how to describe it, so I did it through like showing her what happened, drawing it*’–M001. Another participant described finding constructing a letter to someone involved in the trauma a helpful aspect of the therapy: ‘*I kind of liked, erm, doing like the meditation and letter writing cas I'd never done that before and it did kind of help, yeah*’*–*F009.

There was a strong sense that the positive relationship with both their therapist helped young people feel able to talk about their traumatic experiences openly and honestly. The therapist approach was described as non‐judgmental, understanding and easy‐going by the young people. Two participants reported that the therapist showing a sense of humour also helped to ease any awkwardness and helped them to feel more comfortable:

Erm, we had lots of fun jokes which was quite nice, being able to have that work, get through the therapy and then going on to talking about jokes, and life, and laundry and fun stuff, it was very much like a good therapist relationship –F005

One participant did describe some negative aspects of working with her therapist, saying she felt her therapist was ‘*harsh*’, reflecting that this led to her feeling at times dismissed, that her therapist ‘*was not on her side*’ and this resulted in her feeling less able to talk about her experiences. However, overall, the findings suggest that participants valued being involved in the decision making about the pace, location, completion of homework tasks and the gender of their therapist.

### Theme 3: Positive changes and increased ability to cope

All participants described positive changes following therapy. The following subthemes describe the young people experiencing more positive emotions and improved self‐esteem, with participants feeling more able to cope with their difficulties, through talking with others and utilising specific strategies.

#### Subtheme 3.1: Changes in themselves and relationships

Many participants experienced positive changes in their emotions (‘*a lot more happier*’, M001) and how they felt about themselves, which included feeling like a stronger person and a sense of acceptance of their identity:

I think I'm finally more myself now, it's like it's helped with me come to realise who I am and that's not going to change, so I'm just going to learn to be me –F003

One of the dominant narratives described by the participants was noticing how their relationships with others (i.e. family, friends and/or partners) had improved, which included less arguments, feeling closer to others, people being more understanding and supportive and new friendships emerging. Nine of the participants described feeling more open with others and felt that they could now talk to other people about their traumatic experiences:

yeah, where I've been used to talking about it with another person I'm not bothered about talking about it now, because where, before I wasn't talking to anyone about what had happened so it was just, like playing on my mind and things –F007

#### Subtheme 3.2: Increased ability to cope

Participants described strategies which had increased their ability to cope with difficult emotions and symptoms, these included grounding techniques, relaxation exercises (e.g. ‘*peaceful place*’, M003), breathing techniques, looking back over timeline work and self‐encouragement (labelled as ‘*tough love*’ and ‘*pep talks*’ by one participant, F005). Those participants who had initially described feeling anger reflected that this had reduced, “*helped keep me calmer and stuff*” M001, and felt more relaxed and able to understand and manage their emotions. It appeared that strategies for controlling anger formed an important part of the therapy for the male participants, who were also younger (aged between 12 and 14 years old), ‘*we tried to find ways of controlling my anger, and–that was pretty much it*’, M002. Whereas the older females appeared to reflect more about the impact of the trauma processing work.

When asked about ending therapy sessions, many of the participants described how the sessions were phased out, reducing from weekly to fortnightly. There was a sense that the participants felt ready to finish (*n* = 8), ‘*I could say I was sad, but I was also satisfied in the sense that it's like there's nothing more now*’, F006, suggesting they were more able to cope without therapeutic input.

To summarise, all participants described positive changes in their emotions, and how they viewed themselves. There was a sense that the young people felt more open within their key relationships and an increased ability to cope, which included utilising strategies they had learned during therapy.

## DISCUSSION

### Summary of findings

This qualitative study explored young people's experience of talking about their traumatic experiences both prior to, and within the context of, an RCT exploring the effectiveness of CT‐PTSD. Using thematic analysis, three key themes were identified, firstly, ‘desire for change’ where young people wanted to get the ‘right help’ for their trauma‐related difficulties and to help other young people with similar difficulties. The second theme ‘journey of becoming able to talk about trauma’, illustrates how previous avoidance of talking about their trauma progressed to the young people becoming more open and being able to make sense of their trauma. It appeared that this journey was facilitated by a positive therapeutic relationship, the use of written tasks and opportunities to be involved in decision‐making regarding their therapy. Lastly, the theme ‘positive changes and increased ability to cope’, details how participants felt more positive about themselves and their relationships with others, and utilised coping strategies.

### Research findings in context

Many of the young people reported that prior to the RCT there was a strong sense of avoiding talking about trauma, which reflects key symptoms of PTSD related to avoidance. Many therapists find working with trauma‐exposed youth difficult and demanding (Allen & Johnson, 2012), and are concerned that talking about trauma may increase the young people's distress leading to re‐traumatisation (Finch et al., 2020). Participants in the current study report that this avoidance of trauma‐focused work led them to feel let down and that their difficulties persisted, even after engaging in other psychological therapies. However, young people in the present study found it beneficial to talk about trauma during the sessions and reported finding psychological trauma‐focused support helpful. While they reported that talking about traumatic experiences was difficult and emotionally draining, over time it became easier and resulted in them feeling accomplished and relieved, providing a sense of empowerment and control over their traumatic experiences. This finding of ‘time‐related diffusion of negative affect’ has been reported several times in the literature, highlighting the robustness of this effect (Carter‐Visscher et al., 2007; Dittmann & Jensen, 2014).

A critical component of CT‐PTSD involves constructing a trauma narrative, where often young people are encouraged to use a variety of creative expressions to tell the story of their trauma (Smith et al., 2010). This component of the therapy, in particular the development of a timeline and the use of drawing, was held in high regard by the participants in the current study, where it was described how this helped to make sense of their traumatic experiences. The usefulness of the timelines adds to the literature that constructing written narratives, for those who have experienced multiple traumatic events and are experiencing complex traumatisation, is likely to be a vital part of trauma‐focused therapies (Ruf et al., 2010; Smith et al., 2010).

An encouraging finding in the present study is that all participants involved in this qualitative study reported positive changes after engaging in CT‐PTSD. Some participants reported an absence of PTSD symptoms, others felt whilst symptoms were still present, they were able to understand and cope with them more effectively. Despite relaxation training not being a central component of CT‐PTSD (Smith et al., 2010), a few participants described finding the use of strategies, such as safe place exercises, helpful in managing their difficulties. It appeared that the younger male participants utilised these strategies, potentially suggesting that younger children may benefit from skills training to help them tolerate higher levels of anxiety and more difficult emotions during therapy.

Participants reported that following CT‐PTSD they felt more open to talking about their traumatic experiences with others, outside of the therapeutic relationship. Helping to establish positive, trusting relationships is an important part of trauma‐focused psychological interventions, especially for young people experiencing complex trauma where relationships have often been disrupted (Cohen et al., 2012). By talking about their experiences with others this may provide opportunity for the young people to feel empowered and helping people to find their voice is key to healing from trauma (Malekoff, 2008; Wise, 2005). These findings corroborate existing literature where traumatised youth highlight the importance of being involved in decision‐making related to their care and that it is crucial to have a positive, trusting relationship to facilitate effective trauma treatment (Graham & Johnson, 2021).

### Strengths and limitations

Whilst attempts were made to contact two participants who had dropped out of the trial, neither agreed to participate in an interview. It is likely that the views presented in this study do not reflect those of the participants who may have had more unhelpful experiences and therefore this may limit the conclusions that can be drawn. Furthermore, the interviewers were either former or current trial managers which may have shaped the interviews and analysis. To manage this, it was ensured that the interviewers had not been involved in participants' research assessments and researcher diaries and audio recordings were reviewed in order to consider researcher positioning throughout. The current qualitative study was embedded within a pragmatic, real world RCT, which is both a strength and limitation, as it does reflect, to some extent, routine clinical practice within NHS CAMHS. However, this is also a limitation as it can be harder to draw out more information about the process and implementation of the CT‐PTSD treatment as the focus of this qualitative sub study was to capture the experience of young people engaged in CT‐PTSD. The main research paper will provide more insights into the process related mechanisms, for example, treatment credibility, however future qualitative research may wish to focus on also embedding more process related data and analysis into their design.

### Clinical implications and future research

This study contributes to the finding that young people find it helpful to talk about their traumatic experiences, and therefore highlights the need for therapists to address trauma. It is worth noting that therapists involved in delivering CT‐PTSD were offered additional training and supervision as part of involvement in the RCT, which is likely to have increased their knowledge and confidence to deliver trauma‐informed therapy. It will be key for services to provide appropriate training and supervision to ensure the provision of effective trauma focused interventions, with careful consideration to providing emotional support to alleviate anxieties and emotional burden, and to increase the confidence of therapists (Finch et al., 2020). Future research would benefit from investigating current therapist's views of the training and supervision in CAMHS to identify potential training‐practice gaps.

Young people valued therapist flexibility, which included involvement in the decision‐making, such as providing opportunities for them to make decisions about which traumatic memories to discuss. This has also been found in previous studies where participants were most satisfied when they felt they were not being pressured by therapists to talk about traumatic incidents (Dittmann & Jensen, 2014). It appeared that this collaborative and transparent way of working, alongside the supportive, non‐judgmental attitude of the therapists and researchers, helped to facilitate a positive and trusting therapeutic relationship. These decision‐making opportunities are likely to provide much needed control and a sense of empowerment for children and young people who may have experienced traumatic events where they have felt out of control and unsafe. It will be important for clinicians delivering CT‐PTSD as a treatment manual to allow scope for flexibility and adaptability, providing opportunities for the young people to control components of the therapy, in order to meet the individual needs and preferences of their clients.

### Conclusions

For young people with PTSD relating to multiple trauma engaging in CT‐PTSD can be empowering and allows the young people the opportunity to process their traumatic experiences. Participants alluded to previous therapies avoiding talking about their traumatic experiences which led to perpetuation of symptoms and mistrust of professionals. Participants discuss engaging in CT‐PTSD as a journey of becoming able to talk about their traumatic experiences, and an increased ability to cope and talk about their experiences more openly with others outside of the therapeutic relationship. Participants reported feeling more able to open up about their traumatic experiences when trauma‐focused interventions are delivered in the context of a positive therapeutic relationship and when they are involved in decision making throughout the intervention.

## AUTHOR CONTRIBUTIONS

**Leila Allen:** Conceptualization; investigation; writing – original draft; methodology; validation; visualization; writing – review and editing; formal analysis; project administration; data curation. **Andrew Fox:** Writing – review and editing; supervision. **Alexandre Copello:** Writing – review and editing; supervision. **Richard Meiser‐Stedman:** Conceptualization; writing – review and editing; methodology; supervision; funding acquisition. **Nicola Morant:** Writing – review and editing; supervision; conceptualization.

## FUNDING INFORMATION

DECRYPT (‘Delivery of Cognitive Therapy for Young People After Trauma’–randomised controlled trial) is funded by a National Institute for Health Research (NIHR) Career Development Fellowship to RM‐S (CDF‐2015‐08‐073). This current qualitative study was completed as part of a Thesis for a Clinical Psychology Doctorate at the University of Birmingham, the NIHR had no role in the design of the study, or the collection, analysis, and interpretation of data, or in writing the manuscript.

## CONFLICT OF INTEREST STATEMENT

The authors have no conflicts of interest to declare.

## Data Availability

The data that support the findings of this study are available from the corresponding author upon reasonable request.

## References

[papt12558-bib-0001] Allen, B. , & Johnson, J. C. (2012). Utilization and implementation of trauma‐focused cognitive–behavioral therapy for the treatment of maltreated children. Child Maltreatment, 17(1), 80–85. 21875905 10.1177/1077559511418220

[papt12558-bib-0002] Allen, L. , Ashford, P.‐A. , Beeson, E. , Byford, S. , Chow, J. , Dalgleish, T. , Danese, A. , Finn, J. , Goodall, B. , Grainger, L. , Hammond, M. , Humphrey, A. , Mahoney‐Davies, G. , Morant, N. , Shepstone, L. , Sims, E. , Smith, P. , Stallard, P. , Swanepoel, A. , … Meiser‐Stedman, R. (2021). The DECRYPT trial: Study protocol for a phase II randomised controlled trial of cognitive therapy for post‐traumatic stress disorder (PTSD) in youth exposed to multiple traumatic stressors. BMJ Open, 11(7), e047600. 10.1136/bmjopen-2020-047600 PMC8252885 34210731

[papt12558-bib-0003] American Psychiatric Association (Ed.). (2013). The diagnostic and statistical manual of mental disorders (5th ed.). American Psychiatric Association.

[papt12558-bib-0004] Ascienzo, S. , Sprang, G. , & Royse, D. (2022). “My bad experiences are not the only things shaping me anymore”: Thematic analysis of youth trauma narratives. Journal of Child & Adolescent Trauma, 15(3), 741–753. 35958719 10.1007/s40653-021-00431-4 PMC9360376

[papt12558-bib-0005] Bentovim, A. , Cox, A. , Bingley Miller, L. , & Pizzey, S. (2009). Safeguarding children living with trauma and family violence: Evidence based assessment, analysis and planning interventions. Jessica Kingsley.

[papt12558-bib-0006] Braun, V. , & Clarke, V. (2006). Using thematic analysis in psychology. Qualitative Research in Psychology, 3(2), 77–101. ISSN 1478‐0887 Available from. http://eprints.uwe.ac.uk/11735

[papt12558-bib-0007] Braun, V. , & Clarke, V. (2019). Reflecting on reflexive thematic analysis. Qualitative Research in Sport, Exercise and Health, 11(4), 589–597.

[papt12558-bib-0008] Carter‐Visscher, R. M. , Naugle, A. E. , Bell, K. M. , & Suvak, M. K. (2007). Ethics of asking trauma‐related questions and exposing participants to arousal‐inducing stimuli. Journal of Trauma & Dissociation, 8(3), 27–55. 10.1300/J229v08n03_03 18032343

[papt12558-bib-0009] Cohen, J. A. , Mannarino, A. P. , Kliethermes, M. , & Murray, L. A. (2012). Trauma‐focused CBT for youth with complex trauma. Child Abuse & Neglect, 36, 528–541. 22749612 10.1016/j.chiabu.2012.03.007 PMC3721141

[papt12558-bib-0010] Downey, C. , & Crummy, A. (2022). The impact of childhood trauma on children's wellbeing and adult behavior. European Journal of Trauma & Dissociation, 6(1), 100237.

[papt12558-bib-0011] Dittmann, I. , & Jensen, T. K. (2014). Giving a voice to traumatized youth—Experiences with trauma‐focused cognitive behavioral therapy. Child Abuse & Neglect, 38(7), 1221–1230. 24367942 10.1016/j.chiabu.2013.11.008

[papt12558-bib-0012] Eastwood, O. , Peters, W. , Cohen, J. , Murray, L. , Rice, S. , Alvarez‐Jimenez, M. , & Bendall, S. (2021). “Like a huge weight lifted off my shoulders”: Exploring young peoples' experiences of treatment in a pilot trial of trauma‐focused cognitive behavioral therapy. Psychotherapy Research, 31(6), 737–751. 33283674 10.1080/10503307.2020.1851794

[papt12558-bib-0013] Finch, J. , Ford, C. , Grainger, L. , & Meiser‐Stedman, R. (2020). A systematic review of the clinician related barriers and facilitators to the use of evidence‐informed interventions for post traumatic stress. Journal of Affective Disorders, 263, 175–186. 31818775 10.1016/j.jad.2019.11.143

[papt12558-bib-0014] Gilbert, R. , Widom, C. S. , Browne, K. , Fergusson, D. , Webb, E. , & Janson, S. (2009). Burden and consequences of child maltreatment in high‐income countries. The Lancet, 373(9657), 68–81. 10.1016/S0140-6736(08)61706-7 19056114

[papt12558-bib-0015] Graham, S. , & Johnson, D. R. (2021). Trauma therapy: Exploring the views of young people in care. Residential Treatment for Children & Youth, 38(1), 2–18.

[papt12558-bib-0016] Horowitz, M. , Wilner, N. , & Alvarez, W. (1979). Impact of event scale: A measure of subjective stress. Psychosomatic Medicine, 41(3), 209–218. 472086 10.1097/00006842-197905000-00004

[papt12558-bib-0017] King, N. (2004). Using templates in the thematic analysis of text. In C. Cassell & G. Symon (Eds.), Essential guide to qualitative methods in organizational research (pp. 257–270). Sage.

[papt12558-bib-0018] Lewis, S. J. , Arseneault, L. , Caspi, A. , Fisher, H. L. , Matthews, T. , Moffitt, T. E. , & Danese, A. (2019). The epidemiology of trauma and post‐traumatic stress disorder in a representative cohort of young people in England and Wales. The Lancet Psychiatry, 6(3), 247–256. 30798897 10.1016/S2215-0366(19)30031-8 PMC6384243

[papt12558-bib-0019] Malekoff, A. (2008). Transforming trauma and empowering children and adolescents in the aftermath of disaster through group work. Social Work With Groups, 31(1), 29–52. 10.1300/J009v31n01_04

[papt12558-bib-0020] Merikangas, K. R. , He, J. P. , Burstein, M. , Swanson, S. A. , Avenevoli, S. , Cui, L. , Benjet, C. , Georgiades, K. , & Swendsen, J. (2010). Lifetime prevalence of mental disorders in U.S. adolescents: Results from the National Comorbidity Survey Replication–Adolescent Supplement (NCS‐A). Journal of the American Academy of Child and Adolescent Psychiatry, 49(10), 980–989. 20855043 10.1016/j.jaac.2010.05.017 PMC2946114

[papt12558-bib-0021] Morrow, V. , & Richards, M. (1996). The ethics of social research with children: An overview 1. Children & Society, 10(2), 90–105.

[papt12558-bib-0022] National Institute for Health and Care Excellence . (2018). Post‐traumatic stress disorder. (NICE Clinical Guideline 116). https://www.nice.org.uk/guidance/ng116/ 31211536

[papt12558-bib-0023] Radford, L. , Corral, S. , Bradley, C. , Fisher, H. , Bassett, C. , Howat, N. , & Collishaw, S. (2011). Child abuse and neglect in the UK today. In NSPCC.

[papt12558-bib-0024] Ruf, M. , Schauer, M. , Neuner, F. , Catani, C. , Schauer, E. , & Elbert, T. (2010). Narrative exposure therapy for 7‐to 16‐year‐olds: A randomized controlled trial with traumatized refugee children. Journal of Traumatic Stress, 23(4), 437–445. 20684019 10.1002/jts.20548

[papt12558-bib-0025] Salloum, A. , Dorsey, C. S. , Swaidan, V. R. , & Storch, E. A. (2015). Parents' and children's perception of parent‐led trauma‐focused cognitive behavioral therapy. Child Abuse & Neglect, 40, 12–23. 25534316 10.1016/j.chiabu.2014.11.018

[papt12558-bib-0026] Smith, P. , Yule, W. , & Clark, D. M. (2010). Post‐traumatic stress disorder: Cognitive therapy with children and young people. Routledge.

[papt12558-bib-0027] Wise, J. B. (2005). Empowerment practice with families in distress. Columbia University Press.

[papt12558-bib-0028] World Health Organization . (2022). International classification of diseases, eleventh revision (ICD‐11). https://icd.who.int/

